# Editorial: Exploring obesity risk, prevention, and research innovation in the first 2000 days of life

**DOI:** 10.3389/fendo.2022.1040328

**Published:** 2022-10-07

**Authors:** Sarah Taki, Li Ming Wen, Gengsheng He

**Affiliations:** ^1^ Sydney School of Public Health, Faculty of Medicine and Health, The University of Sydney, Sydney, NSW, Australia; ^2^ Population Health Research & Evaluation Hub, Sydney Local Health District, Sydney, NSW, Australia; ^3^ National Health and Medical Research Council (NHMRC) Centre of Research Excellence in the Early Prevention of Obesity in Childhood (EPOCH), The University of Sydney, Sydney, NSW, Australia; ^4^ School of Public Health, Fudan University, Shanghai, China

**Keywords:** obesity, risk, prevention, research innovation, first 2000 days, editorial

Research in the first 2000 days of life, particularly in obesity prevention, has increased over the past few decades due to the globally increasing rates of overweight and obesity among young children in both developed and developing countries. (1, 2) Several reviews and epidemiological studies have shown obesity-related behaviors like poor diet quality, low physical activity, high sedentary behaviors and poor sleep are established early and track from early childhood to later childhood and adolescence and then into adulthood. (3–5) It is therefore imperative to consider interventions in the first 2000 days of life, a critical period for development and the learning of these behaviors, and when biology is most amenable to change thus increasing the likelihood to have sustained effects on health.

More research is therefore needed to understand effective interventions on behavioural outcomes related to obesity prevention and the contributors of overweight and obesity. With this in mind we selected the theme for this Research Topic. We wish to highly commend the authors for their well written papers delivering a range of intervention approaches to promote healthy behaviors and healthy weight gain, highlighting challenges and solutions to interventions approaches and highlighting a range of issues related to some potentially proposed solutions of obesity prevention such as non-nutritive sweeteners. In this editorial we wish to highlight several exciting and promising studies on obesity prevention and research innovation on the first 2000 days of life.

The paper by Laws et al. is a study protocol that describes the first known study to evaluate a scale up of an evidence based early prevention program in Victoria, Australia, INFANT, which is being implemented as an effectiveness-implementation hybrid trial. INFANT supports parents with increasing their knowledge and skills on promoting lifestyle behaviors promote healthy weight gain, through group sessions delivered to first time parents. Since the efficacy trial (2008-2012), a small scale translation was implemented across 8 Local Government Areas (LGA) in Victoria (2011-2016) and now (2019-2024) a state-wide (n=79 LGA’s) scale up approach is implemented. This study will involve engaging and collaborating with policy makers and various services, deliver training to various health professionals to implement INFANT within their practice. Implementation outcomes, process measures and effectiveness outcomes will be measured using qualitative and quantitative measures. This innovative study will inform future scale-up of public health interventions, globally.

An innovative study by Seidler et al. based on a previously published protocol about identifying collaborative and scalable solutions to deliver effective early childhood obesity prevention interventions highlights key challenges experienced in early childhood obesity prevention interventions which make it hard for these interventions to be scaled up. Challenges include understanding the indirect causal pathways of interventions which lead to desired outcomes, the complexity and heterogeneity of interventions limits the translation and synthesis of the evidence produced, promoting behavior change on a population level often lacks cultural responsiveness, intervention effects often fade-out if not continued due to rapid growth in children and lack of feasibility to integrate prevention interventions into services due to cost-effectiveness. Six potential solutions have been proposed to address these challenges ranging from stakeholder and consumer engagement and study design, collaboration with researchers working in similar spaces and consistent intervention reporting.

The paper by Ekambareshwar et al. examined participant engagement with a 6-staged telephone-based health promotion program, Healthy Beginnings delivered from third trimester to 12 months of the child’s age. The study used participant engagement data with the telephone support program (telephone calls answered of the 6 calls) as well as demographic characteristic data collected during the baseline survey (third trimester). Participants’ were categorized into three engagement levels (high, medium, low) based on the number of calls answered across the 6 stages. The study found that participants who were born in Australia, with a higher house-hold income, employed, spoke English at home and older than 30 years were significantly more engaged with the telephone support program.

The paper by Shum and Georgia present a review that focuses on and argues that the negative effects of non-nutritive sweeteners (NNS), a substitute for table sugar, outweighs the positive. The review summarizes the literature providing opposing findings within studies demonstrating the benefits of adults and children consuming NNS to prevent and manage weight. However, it highlights that there is a gap in the literature which reports on the long term effects that NNS has on children’s weight. The review then delves into the physiological mechanisms that contributes to the negative impact that NNS has on the body arguing that it can cause an increase caloric consumption, effect gut microbiota and lead to pancreatic endocrine dysfunction among children, particularly during a critical developmental period.

In addition, Lin et al. reported the use of machine learning to identify metabolic subtypes of obesity. This was a proof-of-concept study that provided evidence of feasibility of using machine learning in obesity classification, which has a great potential to guide therapeutic planning and decisions for different subtypes of obesity. We believe the use of machine learning technique in obesity classification can significantly improve clinical practice for better patient care and treatment.

With the success of this edition, we are excited to announce the launch of Volume II of this Research Topic. Please access the Journal website for submitting papers.

## Author contributions

ST prepared a first draft. LW and GH revised and finalized the editorial. All authors contributed to the article and approved the submitted version.

## Conflict of interest

The authors declare that the research was conducted in the absence of any commercial or financial relationships that could be construed as a potential conflict of interest.

## Publisher’s note

All claims expressed in this article are solely those of the authors and do not necessarily represent those of their affiliated organizations, or those of the publisher, the editors and the reviewers. Any product that may be evaluated in this article, or claim that may be made by its manufacturer, is not guaranteed or endorsed by the publisher.
